# CEMIG: prediction of the cis-regulatory motif using the de Bruijn graph from ATAC-seq

**DOI:** 10.1093/bib/bbad505

**Published:** 2024-01-06

**Authors:** Yizhong Wang, Yang Li, Cankun Wang, Chan-Wang Jerry Lio, Qin Ma, Bingqiang Liu

**Affiliations:** School of Mathematics, Shandong University, Jinan, 250100, China; Department of Biomedical Informatics, College of Medicine, The Ohio State University, Columbus, OH, 43210, USA; Department of Biomedical Informatics, College of Medicine, The Ohio State University, Columbus, OH, 43210, USA; Department of Biomedical Informatics, College of Medicine, The Ohio State University, Columbus, OH, 43210, USA; Pelotonia Institute for Immuno-Oncology, The James Comprehensive Cancer Center, The Ohio State University, Columbus, OH, 43210, USA; Department of Biomedical Informatics, College of Medicine, The Ohio State University, Columbus, OH, 43210, USA; Pelotonia Institute for Immuno-Oncology, The James Comprehensive Cancer Center, The Ohio State University, Columbus, OH, 43210, USA; School of Mathematics, Shandong University, Jinan, 250100, China

**Keywords:** motif finding, chromatin accessibility, algorithms, graph theory, cluster analysis

## Abstract

Sequence motif discovery algorithms enhance the identification of novel deoxyribonucleic acid sequences with pivotal biological significance, especially transcription factor (TF)-binding motifs. The advent of assay for transposase-accessible chromatin using sequencing (ATAC-seq) has broadened the toolkit for motif characterization. Nonetheless, prevailing computational approaches have focused on delineating TF-binding footprints, with motif discovery receiving less attention. Herein, we present Cis rEgulatory Motif Influence using de Bruijn Graph (CEMIG), an algorithm leveraging de Bruijn and Hamming distance graph paradigms to predict and map motif sites. Assessment on 129 ATAC-seq datasets from the Cistrome Data Browser demonstrates CEMIG’s exceptional performance, surpassing three established methodologies on four evaluative metrics. CEMIG accurately identifies both cell-type-specific and common TF motifs within GM12878 and K562 cell lines, demonstrating its comparative genomic capabilities in the identification of evolutionary conservation and cell-type specificity. In-depth transcriptional and functional genomic studies have validated the functional relevance of CEMIG-identified motifs across various cell types. CEMIG is available at https://github.com/OSU-BMBL/CEMIG, developed in C++ to ensure cross-platform compatibility with Linux, macOS and Windows operating systems.

## INTRODUCTION

Regulatory proteins, such as transcription factors (TFs) and ribonucleic acid (RNA)-binding proteins, modulate transcriptional and post-transcriptional processes through deoxyribonucleic acid (DNA) and RNA interactions, respectively [1–11]. The accurate prediction of TF motifs through chromatin immunoprecipitation assay using sequencing (ChIP-seq) is essential, offering insights into the regulatory mechanisms that initiate and control gene expression. ChIP-seq’s effectiveness in motif discovery is limited by its dependency on specific antibodies and the need for large-cell quantities, resulting in potential bias and reduced resolution. In contrast, assay for transposase-accessible chromatin using sequencing (ATAC-seq) circumvents these issues by directly sequencing accessible chromatin, providing a clearer view of motif sites [12].

Over the past decade, ATAC-seq has been leveraged to infer where TFs were bound (i.e. footprinting), providing insights into the regulatory elements that are active in the particular cell line being studied. Although footprinting approaches excel in delineating open chromatin regions and suggesting potential sites for TF activity within ATAC-seq data, it does not possess the capability to directly predict specific motif sites [13–16].

For precise motif prediction, it is essential to apply specialized motif discovery algorithms (such as BioProspector [17], MEME [18], STREME [19] and XXmotif [20]) to the footprints delineated by chromatin footprinting techniques. BioProspector utilizes a Gibbs sampling-based approach to detect overrepresented, conserved motifs across a set of DNA sequences by iteratively refining motif predictions against a background model until convergence [17]. Similar to Gibbs sampling-based methods, MEME applies an expectation–maximization algorithm to discover recurrent and statistically significant motifs within unaligned nucleotide sequences by optimizing motif occurrence probabilities in an iterative refinement process [18]. STREME methodically identifies enriched motifs in DNA sequences by employing an efficient search algorithm that combines rapid convergence with sensitivity to detect even weakly conserved motifs within a comprehensive candidate space [19]. XXmotif leverages a pattern-based approach to systematically enumerate and evaluate candidate motifs in unaligned nucleotide sequences, employing a discriminative optimization strategy that contrasts foreground sequences with a background model to identify statistically significant motifs [20].

However, motif discovery algorithms for ChIP-seq data are predicated on the premise that binding motifs are present at least once within a substantial proportion of peaks. This assumption, however, does not necessarily hold for ATAC-seq footprints, where the distribution and recurrence of motif sites may not align with this pattern of enrichment. Motif inference from ATAC-seq grapples with two principal challenges: first, ATAC-seq footprints suggest potential motif sites without providing conclusive evidence of actual TF occupancy; second, the discernment of specific motif signals is confounded by the presence of numerous non-specific open chromatin regions.

**Figure 1:**
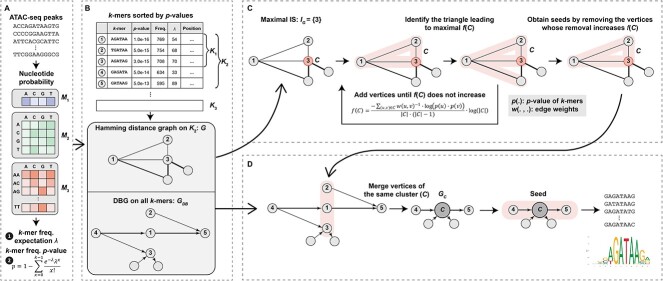
Illustration of the CEMIG framework. (**A**) Determines the $P$ values of *k*-mers in background data utilizing Markov models. (**B**) Constructs Hamming distance graph ($G$) and DBG (${G}_{\mathrm{DB}}$) graphs using $k$-mers. (**C**) Clusters $k$-mers on $G$ to form ${G}_{\mathrm{C}}$, merging same cluster $k$-mers from ${G}_{\mathrm{DB}}$. (**D**) Identifies motifs via path extension in ${G}_{\mathrm{C}}$.

To mitigate these challenges, we present Cis rEgulatory Motif Influence using de Bruijn Graph (CEMIG) (Figure 1) [21], a novel algorithm leveraging de Bruijn Graph (DBG) formulation. CEMIG amplifies motif signals by clustering $k$-mers on a Hamming distance graph, thereby refining the signal within the noisy backdrop of accessible chromatin. Concurrently, CEMIG prioritizes $k$-mer connectivity on DBG over their individual frequency or composition, facilitating the inference of motifs despite the absence of direct binding evidence. The contributions of this study are succinctly summarized as follows:

1. We present CEMIG, a refined motif discovery framework designed for ATAC-seq data (see Materials and Methods for further technical details). Through the synergistic application of two graph-theoretical models, CEMIG adeptly overcomes the specific challenges of motif identification within ATAC-seq datasets.
2. Our validation on ATAC-seq datasets from the Cistrome Data Browser [22], featuring diverse peak counts, establishes CEMIG’s superior efficacy over existing state-of-the-art methods.
3. CEMIG’s utility is further demonstrated through its application to ATAC-seq datasets from GM12878 and K562 cell lines, where it successfully identifies cell-type-specific and shared TF motifs. This capability is instrumental in elucidating the functional genomic landscape, as evidenced by the enriched gene ontology (GO) terms and KEGG pathways, which reflect the differential gene expression and functionalities across cell types [23, 24].

## MATERIALS AND METHODS

### Data acquisition

We acquired 129 ATAC-seq and 28 ChIP-seq datasets from the Cistrome Data Browser (http://cistrome.org/db/) [22] and ENCODE (https://www.encodeproject.org/) [25], respectively, for benchmarking purposes. The number of peaks within these datasets ranged from 848 to 692,368 for ATAC-seq and from 1,111 to 54,070 for ChIP-seq. To identify cell-type-specific and shared motifs in GM12878 and K562 cell lines, we procured two replicates of ATAC-seq and matching RNA-seq datasets from ENCODE. Comprehensive details of these datasets are provided in Supplementary Tables S1 and S2. For in-depth technological aspects of data preprocessing, consult Supplementary note S1 in the Supplementary materials.

### Algorithm

#### Framework of CEMIG

The CEMIG framework encompasses four stages (Figure 1). Initially, CEMIG evaluates input sequences (footprints) to determine $k$-mer (default $k=6$) *P-*values using a Poisson distribution [26, 27], informed by nucleotide frequencies estimated via zero to 2nd order Markov models (Figure 1A). Subsequently, CEMIG sorts $k$-mers by ascending *P-*values and classifies them into three tiers, which facilitates the construction of the Hamming distance graph and the DBG (Figure 1B). Then, CEMIG proceeds by detecting $k$-mer clusters through graph clustering on the Hamming distance graph, then constructs a secondary directed graph (digraph) by amalgamating vertices from identical clusters in the DBG (Figure 1C). Ultimately, CEMIG forecasts motifs and their respective lengths by extending paths within the digraph (Figure 1D).

#### P-value calculation for $k$-mers

CEMIG stores input sequences in set $S=\left\{{S}_1,\dots, {S}_n\right\}$, each with corresponding lengths ${l}_1,{l}_2,\dots, {l}_n$, and constructs matrices ${M}_1$, ${M}_2$ and ${M}_3$, based on frequencies of substrings with lengths ranging from one to three (Figure 1A). Matrix ${M}_1$ represents the frequency of nucleotides A, C, G and T across the input sequences. Matrix ${M}_2$ quantifies the frequency of each nucleotide following another, while ${M}_3$ details the occurrence probabilities of a nucleotide given preceding dinucleotides. CEMIG calculates the expected frequency, $\mathrm{\lambda} (t)$, of a $k$-mer $t={a}_1{a}_2,\dots, {a}_k$ using matrices ${M}_1$, ${M}_2$ and ${M}_3$. This is given by


(1) \begin{align*} \mathrm{\lambda} (t)&=\mathrm{\lambda} \left({a}_1{a}_2,\dots, {a}_k\right)\nonumber\\ &={M}_1\left({a}_1\right){M}_2\left({a}_2|{a}_1\right){M}_3\left({a}_3|{a}_1{a}_2\right){M}_3\left({a}_4|{a}_2{a}_3\right)\dots\nonumber\\ &\qquad{M}_3\left({a}_k|{a}_{k-2}{a}_{k-1}\right)\sum_{i=1}^n\left({l}_n-k+1\right), \end{align*}

where ${l}_i$ is the length of the $i$-th sequence and $n$ is the total number of sequences. CEMIG calculates the $P$ value for a $k$-mer $t={a}_1{a}_2,\dots, {a}_k$ (having frequency $n(t)$) by employing a Poisson distribution model as follows [26, 27],


(2) \begin{equation*} P(t)=1-\sum_{x=0}^{n(t)-1}\frac{e^{-\mathrm{\lambda} (t)}\mathrm{\lambda} {(t)}^x}{x!}. \end{equation*}

#### Graph model construction

##### $k$ *-mer classification*

CEMIG catalogs and ranks $k$-mers in decreasing order based on their $P$-value’s negative logarithm. It designates the top 100 $k$-mers as the highly significant set (${K}_1$), while the first 50% of $k$-mers form the significant set (${K}_2$). The remaining $k$-mers, constituting the latter half and termed ${K}_3$, represent the insignificant $k$-mer group (Figure 1B).

##### Construction of Hamming distance graph

In CEMIG, a Hamming distance graph, denoted as $G$, is constructed where vertices represent $k$-mers from set ${K}_2$ (refer to Figure 1B). An edge is formed between two vertices if the Hamming distance between the corresponding $k$-mers is less than two, with the edge weight being set to the actual Hamming distance.

##### DBG assembly

CEMIG constructs a DBG denoted as ${G}_{\mathrm{DB}}$, utilizing vertices representing all $k$-mers from the combined sets ${K}_2$ and ${K}_3$ (as depicted in Figure 1B). Directed edges are established between $k$-mer pairs when a $\left(k-1\right)$-nucleotide overlap exists between the suffix of one $k$-mer and the prefix of another. For example, if $k$-mers AGCTAG and GCTAGC share a $\left(k-1\right)$ overlap, a directed edge from AGCTAG to GCTAGC is created, with the edge weight representing the frequency of the concatenated $\left(k+1\right)$-mer, AGCTAGC. This process is repeated for both the original sequences and their reverse complements.

#### Graph clustering on Hamming distance graph

In CEMIG’s clustering process applied to the Hamming distance graph $G$, the procedure comprises two pivotal steps: the identification of the maximum independent set (IS) and the subsequent construction of $k$-mer clusters. This procedure facilitates the categorization of $k$-mers based on their mutational proximity, as depicted in Figure 1C. See Supplementary note S2 in the Supplementary materials for supporting details.

##### IS identification

CEMIG initializes the IS, denoted by ${I}_G$, as an empty set and employs a greedy algorithm to iteratively incorporate $k$-mers from the sorted set ${K}_1$ into ${I}_G$, while preserving the set’s independence within graph $G$. CEMIG may not invariably compute the largest maximum IS, yet it frequently yields an approximation that is deemed satisfactory [28].

##### Graph clustering

CEMIG first arranges the vertices in the IS ${I}_G$ by the descending order of their negative logarithmic $P$ values. For each vertex $v$ in ${I}_G$, CEMIG forms a 𝑘-mer cluster $C$ starting with $v$ and the two neighbors in $G$ that maximize the function $f(C)$ defined as follows:


(3) \begin{equation*} { \begin{array}{c}f(C)=\displaystyle\frac{-\sum_{u,v\in C}w{\left(u,v\right)}^{-1}\cdotp \log \left(p(u)\cdotp p(v)\right)}{\left|C\right|\cdotp \left(\left|C\right|-1\right)}\cdot \log \left(\left|C\right|\right)\end{array}}. \end{equation*}

CEMIG then iteratively adds neighboring vertices to $C$ to increase $f(C)$ and removes vertices if it results in an increase in $f(C)$. All clusters are subsequently ordered by the decreasing value of $f\left(\cdot \right)$.

#### Motif discovery via path extension

CEMIG identifies motifs through a three-step process: digraph reconstruction, path extension and motif refinement, as illustrated in Figure 1D. Refer to Supplementary note S3 in the Supplementary materials for additional details.

##### Digraph reconstruction

CEMIG constructs a directed graph ${G}_C$ by amalgamating vertices within identical clusters of ${G}_{\mathrm{DB}}$ into ‘cluster vertices’, excising non-significant $k$-mers and assigning ${G}_C$ edge weights as the cumulative sum of incident edge weights from the corresponding cluster in ${G}_{\mathrm{DB}}$.

##### Path extension

CEMIG employs a greedy algorithm for path extension in ${G}_C$ by starting with an ‘uncovered’ cluster vertex with the highest $f\left(\cdot \right)$ value and sequentially adding vertices from edges with maximum weight, either upstream or downstream. This process continues until the path reaches the length $\left(18-k\right)$, or three $k$-mer vertices have been added in the same direction. The starting cluster and other cluster vertices on the path are then considered ‘covered’. CEMIG outputs paths and iterates the procedure until all clusters are covered.

##### Motif refinement

CEMIG delineates two occurrence sets: ${O}_1$ from the upstream sub-path including the starting cluster and ${O}_2$ from the downstream equivalent. CEMIG refines motifs and their lengths by assessing overlaps between these sets. A significant overlap ($\frac{\mid{O}_1\cap{O}_2\mid }{\min \left({O}_1,{O}_2\right)}>\frac{1}{2}$) where the intersection constitutes over half the size of the smaller set results in a single motif occurrence set from the entire path. A moderate overlap ($\frac{1}{4}<\frac{\mid{O}_1\cap{O}_2\mid }{\min \left({O}_1,{O}_2\right)}\le \frac{1}{2}$) leads to three distinct motif occurrence sets, each corresponding to ${O}_1$, ${O}_2$ and their intersection. Minimal overlap ($\frac{\mid{O}_1\cap{O}_2\mid }{\min \left({O}_1,{O}_2\right)}\le \frac{1}{4}$) maintains two separate motifs corresponding to ${O}_1$ and ${O}_2$.

#### Evaluation metrics

To assess the effectiveness of various motif discovery methods in classifying DNA sequences into positive (likely bound by TFs) and negative (other) samples, we employed four metrics: precision, specificity, accuracy (ACC) and the area under the precision-recall curve (AUPRC). Precision quantifies the proportion of accurately predicted positive samples among all predicted positives. Specificity measures the ratio of correctly identified negative samples to the total negatives, reflecting the model’s aptitude for recognizing negatives. ACC represents the overall proportion of correct predictions across the sample set. AUPRC, the area under the precision-recall curve, is particularly informative in scenarios with imbalanced positive and negative samples, offering a more sensitive measure than the area under the receiver operating characteristic curve. These metrics were computed for each motif discovery method using predicted and actual labels. The range of all criteria scores is 0 to 1, with higher values indicating superior performance. To assess the proficiency in motif identification on ChIP-seq data, we utilized TOMTOM to compute $Q$-values, thereby quantifying the resemblance between the predicted motifs and those cataloged in the HOCOMOCO v.11 database [29, 30].

## RESULTS

### Evaluation on ATAC-seq data

**Figure 2:**
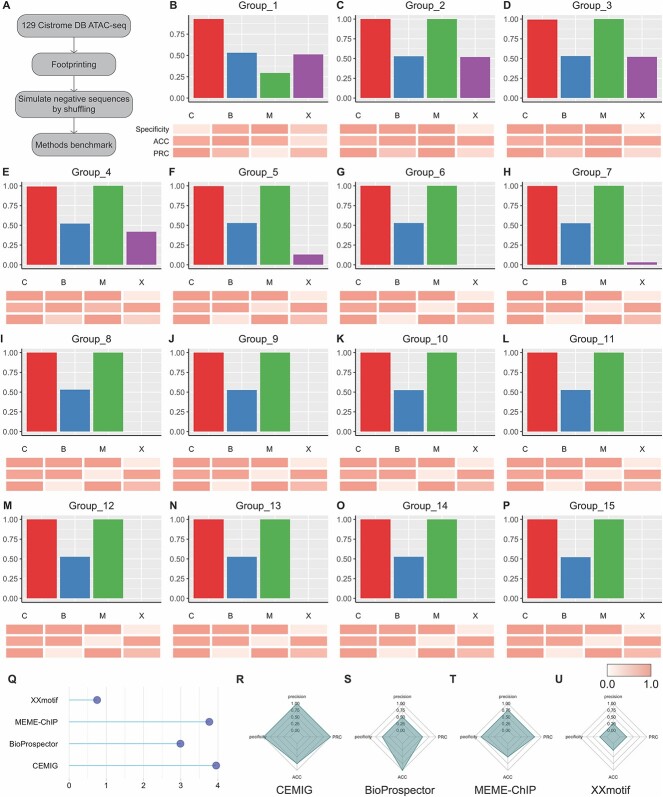
Assessment of CEMIG and other motif discovery methods via Cistrome Data Browser. (**A**) Overview of benchmark data and evaluation approach. (**B**–**P**) Bar charts: Comparative precision across 15 dataset groups, with C, B, M and X representing CEMIG, BioProspector, MEME-ChIP and XXmotif, respectively. Heatmaps: Side-by-side comparison of algorithmic performance in specificity, ACC and AUPRC (normalized across algorithms). (**Q**) Lollipop Chart: Composite scores of each method over 15 groups from 129 datasets. (**R**–**U**) Spider Charts: Average scores of each algorithm in precision, specificity, ACC and AUPRC metrics.

Initially, CEMIG’s performance was evaluated through a binary classification task using 129 human ATAC-seq datasets (refer to Supplementary Table S1 and Figure 2A) [22]. For comparative analysis, we included three leading motif discovery methods: BioProspector [17], MEME-ChIP [6] and XXmotif [20], serving as benchmark controls. While initially designed for ChIP-seq, these methods are also applicable to ATAC-seq footprints, as noted in [15]. The efficacy of each algorithm across these datasets, categorized into 15 incrementally increasing sequence count groups, is detailed in Figure 2B–P. The findings indicate that CEMIG enhances motif discovery performance across the aforementioned 129 ATAC-seq datasets.

The bar plots reveal that CEMIG consistently secures the highest precision in motif discovery across all datasets, with MEME-ChIP closely matching this precision. BioProspector shows stable precision across various dataset sizes and outperforms XXmotif in smaller datasets ($n\le \mathrm{231,120}$). Conversely, XXmotif excels in larger datasets (Figure 2B–F) but frequently fails to produce results within 24 h for these larger sets (Figure 2G–P). In terms of specificity, CEMIG, BioProspector and MEME-ChIP yield similar results, while XXmotif tends to have a higher rate of false negatives (Figure 2B–P). CEMIG and MEME-ChIP show roughly equivalent AUPRC performances across the datasets (Figure 2B–P).

To provide a more intuitive representation of algorithmic performance, we normalized and aggregated four individual metrics into a single composite score (Figure 2Q), revealing that CEMIG and MEME-ChIP outperform BioProspector and XXmotif. Further, we assessed the algorithms based on the ranking of their scores, assigning 1.00 to the top performer and 0.25 to the lowest. Averaging these scores across the 15 groups from the 129 datasets (Figure 2R–U) offered insights into each algorithm’s strengths and weaknesses. While MEME-ChIP exhibits high precision, specificity and AUPRC compared to BioProspector and XXmotif, CEMIG attains the highest average across all metrics. BioProspector notably excels in ACC. XXmotif, hindered by its inability to output results within 24 h, shows suboptimal performance. To corroborate CEMIG’s effectiveness in deriving motif profiles from ATAC-seq data, we conducted a comparative analysis against the HOCOMOCO v.11 database, utilizing TOMTOM for validation (refer to Supplementary Data S1).

### Assessment on ChIP-seq data

We evaluated CEMIG’s ability to detect motifs using 27 ChIP-seq datasets, comparing its performance with MEME-ChIP and DESSO (Supplementary Table S2 and Supplementary Figure S1). The ChIP-seq analysis results indicate that CEMIG effectively identifies motifs for the targeted TFs. These identified motifs show a high degree of similarity to the curated motifs in the HOCOMOCO v.11 database, as measured by TOMTOM $Q$-values, indicating a strong alignment between the detected TF motifs and established motifs in HOCOMOCO v.11.

### Prediction of cell-type-specific and shared motif sites

**Figure 3:**
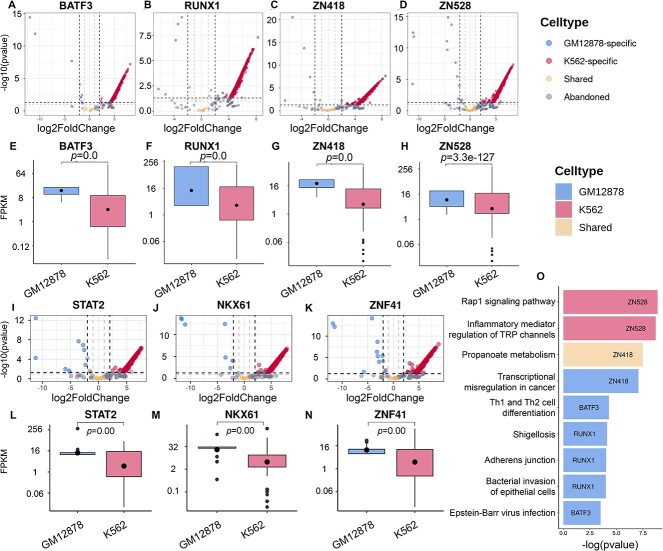
Analysis of cell-type-specific and shared motif sites. (**A**–**D**, **I**–**K**) present the distribution of binding peaks for BATF3, RUNX1, ZN148, ZN528, STAT2, NKX61 and ZNF41. Peaks with a -$Q$value $<0.05$ and a log2 fold-change $<-2$ are categorized as GM12878-specific. Peaks with a -$Q$value $<0.05$ and log2 fold-change $>2$ are K562-specific, while those with a -$Q$value $>0.1$ and an absolute log2 fold-change $<1$ are considered shared. Peaks not classified within the three specified categories are systematically discarded. (**E**–**H**, **L**–**N**) depict cell-type-specific gene expression (RNA-seq) near the binding peaks of these TFs in GM12878 and K562 cells, with Wilcoxon rank sum test $P$-values displayed above the boxes. (**O**) illustrates pathway enrichment for genes nearest to GM12878-specific, K562-specific and shared peaks bound by BATF3, RUNX1, ZN418 and ZN528.

In our study, CEMIG was utilized to discern shared and cell-type-specific motif sites in ATAC-seq data from GM12878 and K562 cells, following the approach in [31] (detailed in Supplementary note S4 in the Supplementary materials). These cell types, with their extensive reference epigenomes, are crucial for understanding epigenetic regulation. CEMIG facilitated the analysis of TF binding to shared and cell-type-specific peaks, exploring their roles in gene regulation and pathway functionalities. Out of 1,700,884 ATAC-seq peaks, we identified 230 GM12878-specific, 32,457 K562-specific and 579 shared peaks involving 55 distinct TFs (Figure 3A–D and I–K). The variation in chromatin accessibility profiles between different cell types reflects their distinct regulatory mechanisms and gene expression patterns.

Our analysis showed that 25 motifs significantly matched ($Q$-values $<0.05$) 21 human reference TF motifs in HOCOMOCO v.11, as determined by TOMTOM (Supplementary Figure S2). Each motif’s sites were categorized as GM12878-specific, K562-specific or shared, based on their corresponding peak locations. We employed the same criteria for -$P$value and log-fold changes as the prior study to categorize cell-type-specific and shared peaks [31]. Despite more K562-specific peaks, GM12878-specific peaks showed wider ranges in both -$P$value and log-fold change (Figure 3A–D and I–K). We further investigated the relationship between cell-type-specific chromatin accessibility and gene expression by correlating genes nearest to each peak with their expression in RNA-seq datasets, measured in fragments per kilobase of transcript per million mapped reads (Figure 3E–H and L–N). Owing to generally lower gene expression levels in K562 than in GM12878 [32], we excluded RNA-seq data comparison in K562 to maintain clear distinctions. Our results indicated differential expression of genes linked to cell-type-specific peaks bound by four representative TFs, with -$P$values $<0.05$. Interestingly, despite a greater number of K562-specific peaks, genes nearest to these peaks were significantly underrepresented, possibly due to the higher $P$-values and log2 fold changes of GM12878-specific peaks, despite their fewer numbers.

To elucidate the biological functions of identified gene sets, we conducted functional genomics analyses, assessing their enrichment against GO terms and KEGG pathways (refer to Supplementary note S5 in the Supplementary materials for additional details) (Figure 3O and Supplementary Data S2). GM12878 cells, derived from B-cells transformed by Epstein–Barr virus (EBV), serve as a model for studying gene regulation, complex signaling networks and the epigenetic basis of cancer progression. Cancer is characterized by altered signaling pathways and gene expression, leading to uncontrolled growth, apoptosis evasion and metastatic potential [33, 34]. Our focus included TFs such as BATF3, a member of the AP-1 family predominant in dendritic cells [35]. BATF3, induced by the NF-κB pathway, regulates EBV gene expression in GM12878 cells [36, 37], suggesting its role in EBV-related diseases [38]. Despite being atypical in B-lymphoblastoid cells like GM12878, BATF3’s low-level expression may influence the antibody response in EBV-infected B cells [38]. RUNX1, expressed in various hematopoietic lineages, is pivotal for hematopoiesis and immune responses [39], including neutrophil activation and macrophage suppression [40, 41]. It is crucial for B cell homeostasis and differentiation [42, 43]. Genes near GM12878-specific, K562-specific and shared peaks bound by ZN418 were enriched in cancer transcriptional misregulation and propanoate metabolism pathways [44]. ZN418, highly expressed in GM12878 as per the ENCODE project [25], may influence cancer progression through its downregulation [44]. While no direct evidence links ZNF418 with propanoate metabolism in these cells, ZNF proteins regulate genes in lipid metabolism and hypoxia response [45, 46]. Finally, ZN528, or ZNF protein 528, is implicated in gene expression regulation, cell proliferation and differentiation [47]. Its role in activating Rap1 signaling in K562 cells is crucial for their proliferation and survival [48, 49]. ZN528 also regulates IL-1 receptor expression, central to inflammatory responses [50].

## CONCLUSION

The computational prediction of motifs from ATAC-seq data has been less explored compared to ChIP-seq, which identifies DNA fragments bound by specific TFs but requires prior knowledge of these TFs. While existing computational methods for ATAC-seq motif analysis show promising performance, they mainly focus on footprinting rather than *de novo* motif prediction and site identification. This manuscript introduces CEMIG, a novel algorithm based on the DBG model for motif prediction. CEMIG was thoroughly evaluated and benchmarked against three established motif-finding tools: BioProspector, MEME-ChIP and XXmotif. We also compared CEMIG-detected motifs with reference motifs in the HOCOMOCO v.11 database using TOMTOM, finding significant similarity (as indicated by -$Q$values) with the reference motifs. Employing CEMIG, we predicted motif sites on GM12878-specific, K562-specific and shared peaks. This revealed differential gene expression near cell-type-specific motif sites. Functional genomics analysis of these gene sets highlighted distinct GO terms and pathways for different cell types and TFs, underscoring CEMIG’s utility and versatility in genomic and epigenomic research.

**Key Points**

- The paper unveils CEMIG, a novel algorithm for predicting transcription factor (TF) binding sites.
- CEMIG emphasizes motif identification in ATAC-seq, an area previously underexplored.
- Using 129 ATAC-seq datasets, CEMIG outperforms three established methodologies.
- CEMIG expertly identifies both specific and shared TF motifs in GM12878 and K562 cells.

## Supplementary Material

Supp-material_bbad505

Supp-data-S1-CEMIG-logos_bbad505

Supp-data-S2-Functional-genomics-analyses_bbad505

## Data Availability

The source code of CEMIG can be found at https://github.com/OSU-BMBL/CEMIG. The data sources used in this paper are reported in the section ‘Materials and Methods’. All of the datasets generated in this study are available upon request.

## Author Biographies

**Yizhong Wang** is currently a doctoral candidate at the Shandong University’s School of Mathematics, specializing in single-cell multi-omics analysis and algorithmic design.

**Yang Li** holds a postdoctoral fellowship in the Department of Biomedical Informatics at The Ohio State University, where his research focuses on algorithm development, gene regulatory networks and neuroscientific methodologies.

**Cankun Wang** serves as a biostatistics analyst at the Ohio State University’s Department of Biomedical Informatics, with a concentration on the development of databases and web servers.

**Chan-Wang Jerry Lio** is an assistant professor at the Ohio State University’s Infectious Diseases Institute, with expertise in the adaptive immune response to infectious agents.

**Qin Ma**, a professor in the Department of Biomedical Informatics at the Ohio State University, conducts research spanning gene regulation, single-cell multi-omics and deep learning applications.

**Bingqiang Liu**, a professor at the Shandong University’s School of Mathematics, is renowned for his work in algorithmic design, deep learning and sequence analysis.
