# Supplementary material for: CEMIG: prediction of the cis-regulatory motif using the de Bruijn graph from ATAC-seq
Source: Brief Bioinform. 2024 Jan 6;25(1):bbad505. doi: 10.1093/bib/bbad505 (PMC10772951; doi:10.1093/bib/bbad505)
Supplement: Supp-material_bbad505 [file supp-material_bbad505.pdf]

Supplementary materials of

CEMIG: Prediction of the *cis*-regulatory motif using the de Bruijn graph from ATAC-seq

Yizhong Wang[1,†], Yang Li[2,†], Cankun Wang[2], Chan-Wang Jerry Lio[2,3], Qin Ma[2,3,*], Bingqiang Liu[1,*]

[1]School of Mathematics, Shandong University, Jinan, 250100, China, [2]Department of Biomedical Informatics, College of Medicine, The Ohio State University, Columbus, OH, 43210, USA, [3]Pelotonia Institute for Immuno-Oncology, The James Comprehensive Cancer Center, The Ohio State University, Columbus, OH, 43210, USA.

*To whom correspondence should be addressed.

[†]The authors wish it to be known that, in their opinion, the first two authors should be regarded as Joint First Authors.

- **Supplementary note S1: Data preprocessing**
- **Supplementary note S2: Graph clustering on Hamming distance graph**
- **Supplementary note S3: Prediction of potential motifs via path extension**
- **Supplementary note S4: Discovery of cell-type-specific motif**
- **Supplementary note S5: Transcriptional and functional genomics analysis**
- **Supplementary Fig. S1. Benchmark motif prediction results on ChIP-seq datasets**
- **Supplementary Fig. S2. Motif logos on GM12878 and K562**
- **Supplementary Table S1. Peak count in ATAC-seq datasets for benchmarking**
- **Supplementary Table S2. Peak count in ChIP-seq datasets for benchmarking**

Supplementary note S1: Data preprocessing

We acquired 129 ATAC-seq datasets in browser extensible data (BED) format from Cistrome Data Browser (http://cistrome.org/db/) and 28 ChIP-seq datasets from ENCODE (https://www.encodeproject.org/), detailed in **Supplementary Tables S1 and S2**. FASTA files for each dataset were generated from BED files using BEDTools v.2.29.1 (https://bedtools.readthedocs.io/en/latest/index.html) with the command: **bedtools getfasta -fi <input FASTA> -bed <BED> -fo <output FASTA>**. For ATAC-seq data, matched binary alignment map (BAM) files were also downloaded, and HINT-ATAC (https://reg-gen.readthedocs.io/en/latest/) was applied for footprinting analysis using: **rgt-hint footprinting --atac-seq --paired-end --output-prefix=<output prefix> <input BAM> <input BED>**, resulting in footprint files in BED format. ATAC-seq footprints and ChIP-seq peaks were labeled as positive sequences. Negative sequences were simulated using the **fasta-dinucleotide-shuffle** function in MEME Suite v.5.5.4: **fasta-dinucleotide-shuffle -f <input FASTA> -t <negative prefix> > <output FASTA>**, and concatenated with positive sequences into a single FASTA file using the **cat** Shell command.

Supplementary note S2: Graph clustering on Hamming distance graph

CEMIG conducts a two-step clustering on the Hamming distance graph, G, involving maximum independent set (IS) identification and k-mer cluster construction.

Maximum IS identification. CEMIG utilizes a greedy algorithm to approximate the maximum IS:

(1) k-mers in K_1 are sorted in descending order by their negative logarithmic P-values.
(2) The maximum IS, I_G, is initially empty.
(3) Each k-mer, v, in $K_1 \backslash I_G$ is considered individually.
(4) If v is not adjacent to any in I_G, it is included in I_G.
(5) If v is adjacent to any in I_G, it is skipped.
(6) After all k-mers are evaluated, I_G is returned as the maximum IS. This approach ensures I_G maintains independence; though not guaranteed to be maximum, it often yields a close approximation [1].

k-mers cluster construction. CEMIG's k-mer clustering is based on vertices in I_G:

(1) Vertices in I_G are sorted in descending order by their negative logarithmic P-values.

(2) A cluster set, initially empty, is established.

(3) For each vertex v in I_G:

    (a) The current cluster $C = \{v\}$ is initialized.

    (b) Neighbors of v in G, $N_G(v)$, are found.

    (c) Vertex pairs in $N_G(v)$ are added to C to maximize $f(C)$, calculated as:

$$f(C) = \frac{-\sum_{u,v \in C} w(u,v)^{-1} \cdot \log\big(p(u) \cdot p(v)\big)}{|C| \cdot (|C| - 1)} \cdot \log(|C|) \quad (1)$$

    $w(u,v)$ is the Hamming distance between u and v; The weighted density of C is given by $\frac{-\sum_{u,v \in C} w(u,v)^{-1}}{|C| \cdot (|C|-1)}$; $p(u)$ and $p(v)$ are the statistical significances of k-mers in C; $log(|C|)$ favors larger clusters. The objective is to group k-mers with small Hamming distances and P-values, indicative of a motif.

(4) Neighbors are iteratively added to C until $f(C)$ no longer increases.

(5) For all v' in C except v, if removing v' increases $f(\cdot)$, it is removed.

(6) Clusters are outputted in decreasing order of $f(\cdot)$.

Supplementary note S3: Prediction of potential motifs via path extension

CEMIG utilizes a greedy algorithm to extend paths in the clustered graph G_C, operating as follows:

(1) It selects an "uncovered" (defined later) cluster with the highest $f(\cdot)$ value as the initial vertex, starting a path with this single vertex.

(2) CEMIG selects the edge with the greatest weight connecting uncovered vertices to the path's termini in both upstream and downstream directions.

(3) This step is repeated until one of two termination criteria is met:

    (a) The path reaches a length of $(18 - k)$, aligning with the typical maximum motif length for eukaryotic TFs, which is under 19 base pairs.

    (b) Three consecutive k-mer vertices have been added in the same direction.

(4) The longest sub-path that includes only the starting cluster and additional cluster vertices is deemed "covered".

(5) The process outputs the current path and reinitiates at Step (1). If there are no remaining uncovered clusters, all paths identified thus far are output as seeds for further motif refinement.

Supplementary note S4: Discovery of cell-type-specific motif

We acquired ATAC-seq datasets for GM12878, including peaks (BED format) and two replicates (BAM format), using ENCODE accession codes ENCFF470YYO, ENCFF415FEC, and ENCFF646NWY. For K562, peaks and replicates were obtained with codes ENCFF246XXM, ENCFF243FUG, and ENCFF832ZQV. To discern differential motifs between GM12878 and K562 cell types, we executed the following procedures [2]:

(1) Peaks from both cell types were amalgamated, and BEDTools was employed to tally reads within each peak from filtered BAM files.

(2) Optionally, read counts underwent normalization to rectify sequencing depth and peak length biases, adhering to Eq. (2)

$$\text{peak}^i = \frac{M^i}{N \times L^i} \times 10^9 \qquad (2)$$

where M represents the mapped reads for the i-th peak, N is the total mapped reads across all peaks, and L signifies the length of the i-th peak.

(3) DESeq2, typically used for RNA-seq data, was adapted to analyze these normalized counts following established ATAC-seq protocols [3].

(4) Using DESeq2 outputs, peaks with a Q-value < 0.05 and a log2 fold-change beyond $[-2,2]$ were earmarked as cell-type-specific peaks. Conversely, peaks with a Q-value > 0.1 and an absolute log2 fold-change less than 1 were designated as shared peaks. Peaks not meeting these criteria were excluded. Motif sites on cell-type-specific peaks and shared peaks are termed as cell-type-specific motif sites and shared motif sites, respectively. Using motif sites, we construct motif profiles, comprising both cell-type-specific motifs and shared motifs.

Supplementary note S5: Transcriptional and functional genomics analysis of genes near cell-type-specific motif sites

We conducted a comparative analysis of gene expression near cell-type-specific motif sites bound by the same transcription factor (TF) using RNA-seq data from ENCODE (accession code ENCFF028CFE). The gene annotation file in GTF format was obtained from the UCSC Genome Browser (https://hgdownload.soe.ucsc.edu/goldenPath/hg19/bigZips/genes/). Using BEDTools, we identified genes proximal to cell-type-specific motif sites with the command: **`bedtools closest -a <motif sites> -b <gene annotations> > <output BED>`**. Differential expression analysis was performed using DESeq2 v.1.42.0. Subsequently, we identified enriched Gene Ontology (GO) terms and KEGG pathways associated with these genes.

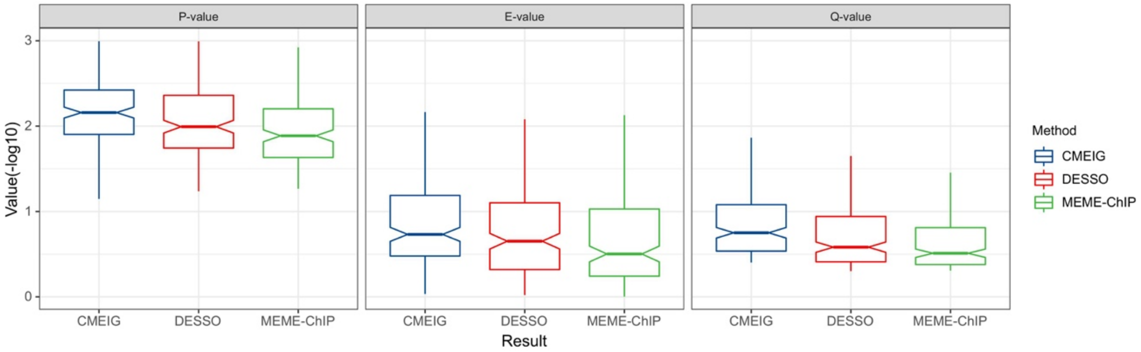

Supplementary Fig. S1. Benchmark motif prediction results of the 27 ChIP-seq datasets in terms of P-values, E-values, and Q-values of motif similarity.

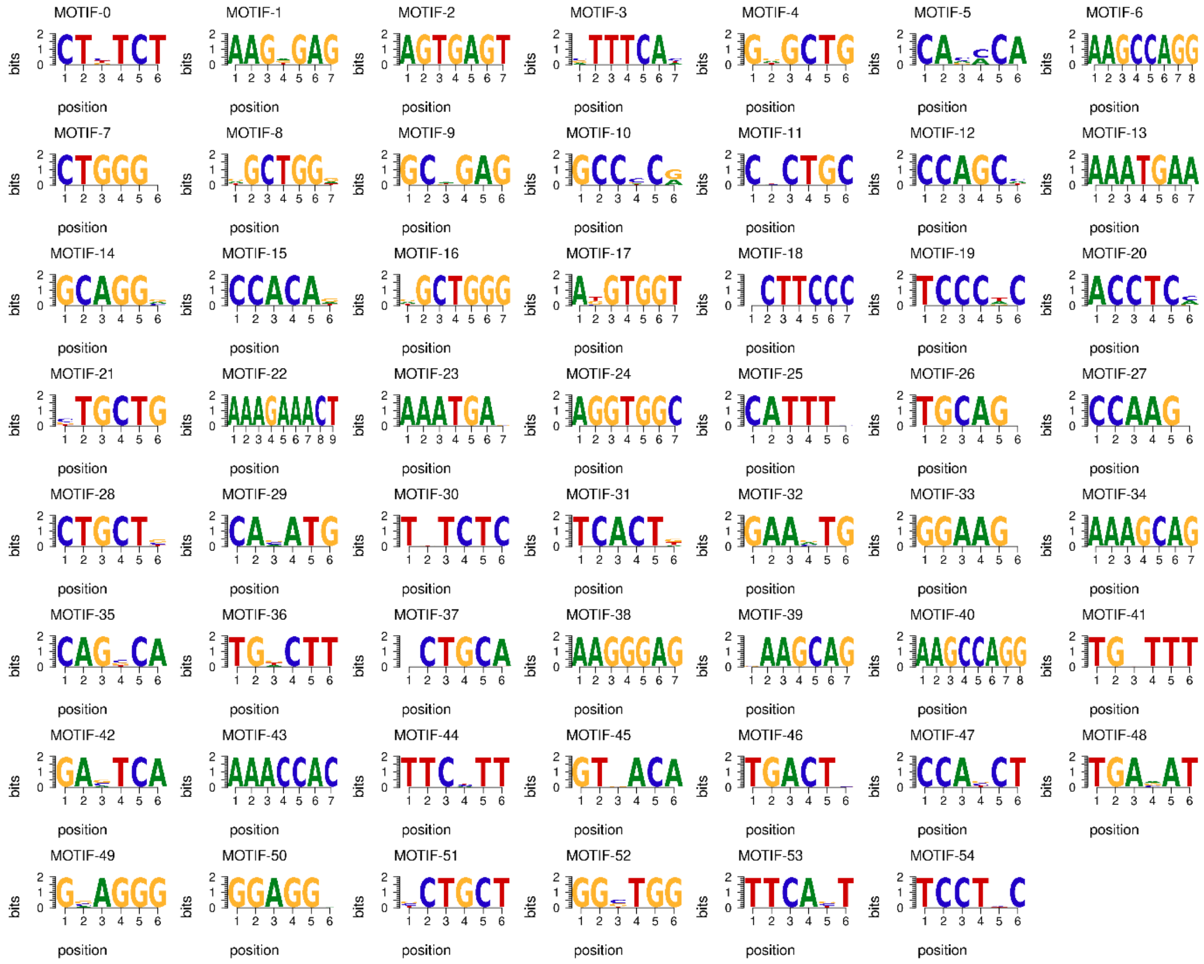

Supplementary Fig. S2. The sequence logos of TF motifs identified on ATAC-seq of GM12878 and K562. Motifs significantly mapped to reference motifs in HOCOMOCO or unknown ones are showcased. The correspondence relationships between discovered motifs and reference ones include: MOTIF-3 (BATF3), MOTIF-10 (AP2D), MOTIF-13 (STAT2), MOTIF-14 (ZIC2), MOTIF-15 (RUNX2), MOTIF-18 (ZN528), MOTIF-19 (NR0B1), MOTIF-21 (ZIC3), MOTIF-22 (STAT2), MOTIF-23 (NKX61), MOTIF-28 (ZIC3), MOTIF-29 (TWST1), MOTIF-32 (IRF4), MOTIF-33 (ELF5), MOTIF-36 (NR2E1), MOTIF-38 (ZNF41), MOTIF-39 (ZN418), MOTIF-41 (ZN418), MOTIF-42 (BACH2), MOTIF-43 (RUNX1), MOTIF-44 (STAT1), MOTIF-46 (BATF), MOTIF-50 (VEZF1), MOTIF-51 (ZIC3), and MOTIF-53 (IRF2).

Supplementary Table S1. Peak count in ATAC-seq datasets for benchmarking

Accession	Peaks	Accession	Peaks	Accession	Peaks	Accession	Peaks
SRR2059154	848	SRR1556325	224820	SRR5800706	361752	SRR5785369	631668
SRR2059152	960	SRR891275	224820	SRR1556371	362544	SRR5196903	644468
SRR2059156	964	SRR2453159	226780	SRR6766910	364132	SRR6955608	650668
SRR3129116	1040	SRR1556339	229812	SRR1556365	365684	SRR5442275	652984
SRR2059155	2096	SRR1947691	230480	SRR6251845	370120	SRR5196904	663008
SRR2059153	2416	SRR6766915	231120	SRR6766914	375896	SRR5800663	666300
SRR2059151	2472	SRR1556341	231960	SRR1556354	378828	SRR5785386	666900
SRR5123142	9076	SRR1556327	234444	SRR6251849	389684	SRR5800664	674268
SRR2059128	13692	SRR891277	234444	SRR1947692	392696	SRR5800665	692368
SRR2059126	14016	SRR1556334	237668	SRR1556368	393264		
SRR2059132	15588	SRR1556372	240324	SRR2453163	407864		
SRR2059130	15632	SRR1556376	241500	SRR5442271	408764		
SRR5123141	18164	SRR1556340	242136	SRR5006007	417308		
SRR2059131	19360	SRR1556350	250072	SRR1556344	419820		
SRR2059127	19856	SRR6766913	254352	SRR5800797	426804		
SRR2059129	23716	SRR1556343	257220	SRR6766916	427380		
SRR1556356	25340	SRR1947693	278528	SRR4436441	430536		
SRR1556377	79120	SRR6251841	279192	SRR1556353	433308		
SRR1556337	94240	SRR1556367	280156	SRR6251843	444796		
SRR1556346	111384	SRR6300362	289044	SRR5800799	455540		
SRR1556335	118512	SRR5442269	289392	SRR5006005	462416		
SRR5785357	122976	SRR5068867	294104	SRR4269916	473964		
SRR1556336	128752	SRR1556332	294332	SRR5800802	482656		
SRR1556366	137352	SRR1556326	294900	SRR1556345	483336		
SRR5626533	138364	SRR891276	294904	SRR6251848	484896		
SRR891280	143276	SRR1556357	298948	SRR6870517	487608		
SRR5785377	148408	SRR5068865	299296	SRR1556329	509780		
SRR1556333	168548	SRR5068866	300356	SRR1556362	511216		
SRR2453162	171964	SRR1556374	309168	SRR5442276	520856		
SRR6766911	185116	SRR6300363	309540	SRR5785364	522376		
SRR1556331	195300	SRR6251846	314784	SRR6766912	545768		
SRR1556338	195504	SRR1556355	319780	SRR1556352	561136		
SRR5442270	201036	SRR1556370	324812	SRR6870516	569924		
SRR1556347	206740	SRR6251844	328256	SRR6730132	574776		
SRR5068863	208780	SRR5442274	334140	SRR2999315	578556		
SRR1556342	210720	SRR1556358	338160	SRR5785370	589184		
SRR1556363	214964	SRR5800798	338692	SRR7275228	599288		
SRR5068861	217984	SRR4269915	345112	SRR1556330	607668		

SRR5068860	218332	SRR5800801	359028	SRR6955605	610308	
SRR5626534	221200	SRR2453161	360736	SRR6730131	630992	

The "Accession" column lists the Sequence Read Archive (SRA) accession codes for benchmarking datasets, and the "Peaks" column indicates the number of sequences in each dataset.

Supplementary Table S2. Peak count in ChIP-seq datasets for benchmarking

Cell line	TF	JASPAR TF ID	JASPAR accession code	Peaks
H1 hESC	FOSL1	MA0477.1	wgEncodeEH001660	1111
GM12878	TR4	MA0504.1	wgEncodeEH000697	1263
H1 hESC	RXRA	MA0512.1	wgEncodeEH001560	1306
H1 hESC	RFX5	MA0510.1	wgEncodeEH001835	1695
GM12878	NFYA	MA0060.2	wgEncodeEH002064	1841
H1 hESC	JUN	MA0488.1	wgEncodeEH001854	2148
K562	ZNF263	MA0528.1	wgEncodeEH000630	3081
K562	USF2	MA0526.1	wgEncodeEH001797	3083
GM12878	E2F4	MA0470.1	wgEncodeEH002867	3440
H1 hESC	POU5F1	MA0142.1	wgEncodeEH001636	3994
K562	SRF	MA0083.2	wgEncodeEH001600	4717
H1 hESC	ATF3	MA0093.2	wgEncodeEH001566	4804
GM12878	SIX5	MA0088.1	wgEncodeEH001542	4839
K562	FOS	MA0476.1	wgEncodeEH000619	7646
H1 hESC	JUND	MA0491.1	wgEncodeEH002023	9550
K562	JUN	MA0488.1	wgEncodeEH000620	9848
K562	GATA2	MA0036.2	wgEncodeEH000683	10648
K562	ETS1	MA0098.2	wgEncodeEH001580	10726
H1 hESC	MAFK	MA0496.1	wgEncodeEH002828	11425
K562	CTCFL	MA0139.1	wgEncodeEH001652	11533
K562	GABP	MA0062.2	wgEncodeEH001604	14393
K562	REST	MA0138.2	wgEncodeEH001638	15849
K562	TBP	MA0108.1	wgEncodeEH001825	17558
GM12878	MEF2A	MA0052.2	wgEncodeEH001565	17605
K562	USF1	MA0093.2	wgEncodeEH001583	18521
K562	EJUND	MA0491.1	wgEncodeEH001211	26674
H1 hESC	CTCF	MA0139.1	wgEncodeEH001649	54070

This table lists the number of identified ChIP-seq peaks for various TFs across various cell lines. Columns display the cell line, the TF name, the corresponding JASPAR TF ID, the JASPAR accession code, and the total peak count for each TF dataset.

References

1. Cormen TH, Leiserson CE, Rivest RL et al. Introduction to algorithms. MIT press, 2022.
2. Zhang Q, Teng P, Wang S et al. Computational prediction and characterization of cell-type-specific and shared binding sites, Bioinformatics 2022;39.

3. Zhang Q, Teng P, Wang S et al. Computational prediction and characterization of cell-type-specific and shared binding sites, Bioinformatics 2022;39:btac798.
