# Supplementary material for: CEMIG: prediction of the cis-regulatory motif using the de Bruijn graph from ATAC-seq
Source: Brief Bioinform. 2024 Jan 6;25(1):bbad505. doi: 10.1093/bib/bbad505 (PMC10772951; doi:10.1093/bib/bbad505)
Supplement: Supp-data-S1-CEMIG-logos_bbad505 [file supp-data-s1-cemig-logos_bbad505.pdf]

Supplementary Data S1. Comparative logos of CEMIG-predicted motifs and HOCOMOCO v.11 curated motifs

Yizhong Wang[1,†], Yang Li[2,†], Cankun Wang[2], Chan-Wang Jerry Lio[2,3], Qin Ma[2,3,*], Bingqiang Liu[1,*]

[1]School of Mathematics, Shandong University, Jinan, 250100, China, [2]Department of Biomedical Informatics, College of Medicine, The Ohio State University, Columbus, OH, 43210, USA, [3]Pelotonia Institute for Immuno-Oncology, The James Comprehensive Cancer Center, The Ohio State University, Columbus, OH, 43210, USA.

*To whom correspondence should be addressed.

†The authors wish it to be known that, in their opinion, the first two authors should be regarded as Joint First Authors.

ELF3

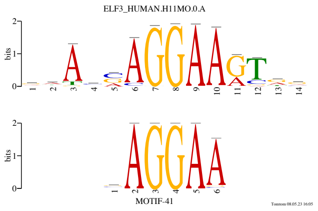

SRR891275

FLI1

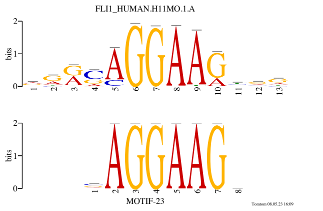

SRR891276

ZNF70

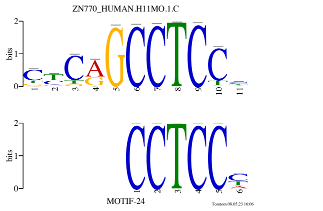

SRR891277

SP1

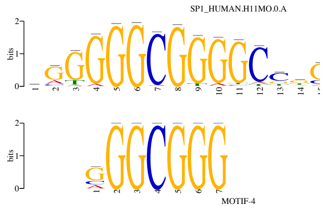

SRR891280

ZN770

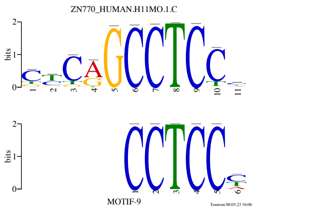

SRR1556341

SP1

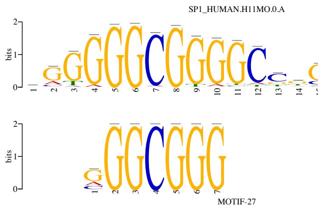

SRR1556342

SP1

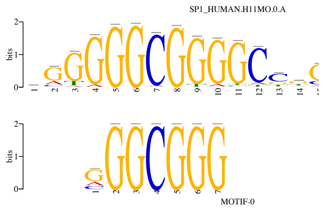

SRR1556343

ZN770

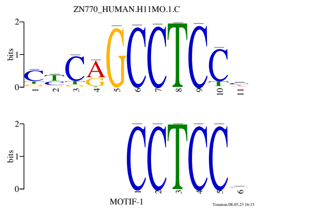

SRR1556345

NFYB

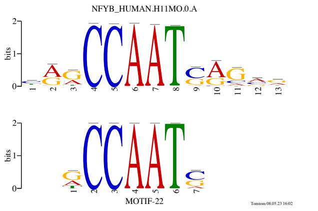

SRR1556346

KLF13

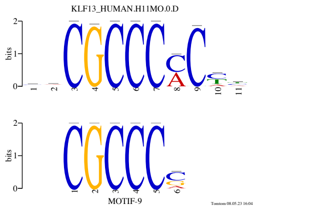

SRR1556347

ZN770

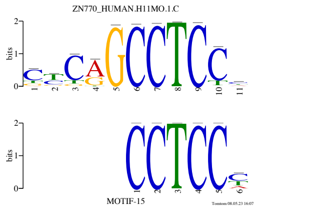

SRR1556350

FLI1

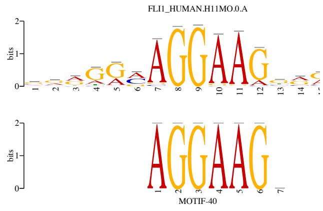

SRR1556353

ZN770

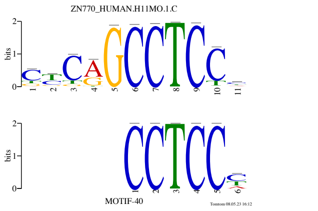

SRR1556354

SP1

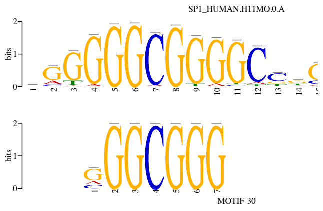

SRR1556355

ZN770

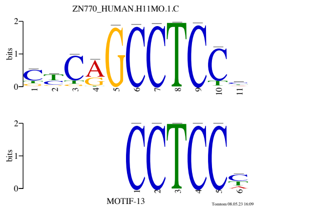

SRR1556357

ZN770

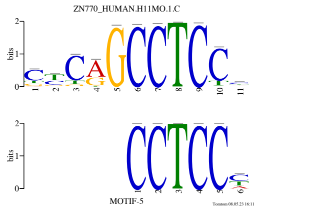

SRR1556358

ELF3

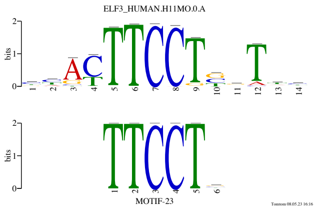

SRR1556362

ZN770

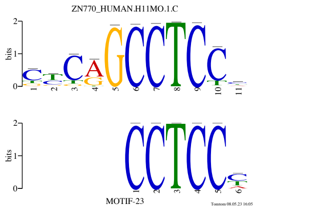

SRR1556363

ZN770

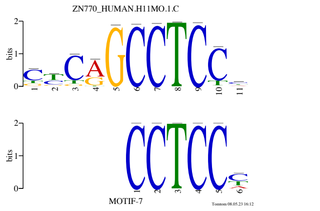

SRR1556365

SP2

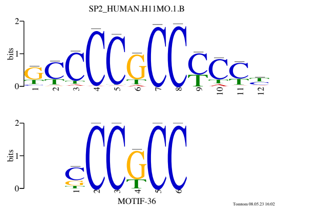

SRR1556366

FLI1

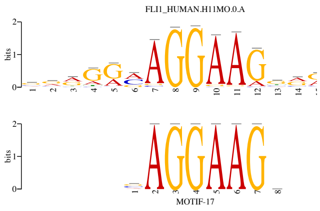

SRR1556367

ZN586

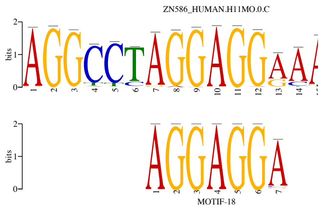

SRR1556368

ZN770

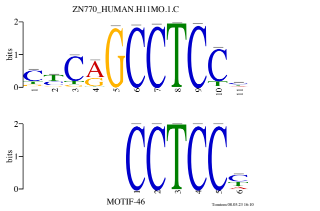

SRR1556370

FLI1

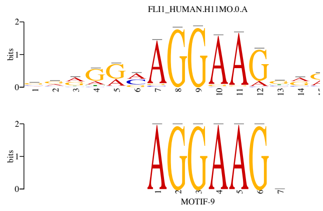

SRR1556371

SP1

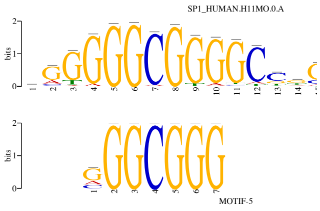

SRR1556372

ZN770

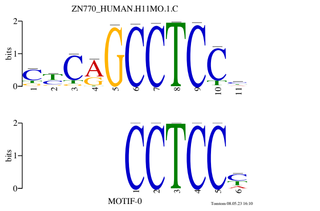

SRR1556374

SP1

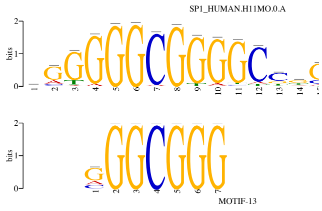

SRR1556376

NFYA

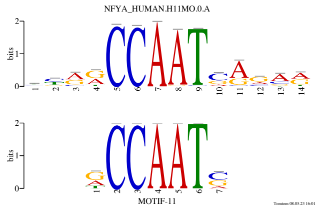

SRR1556377

CR3LI

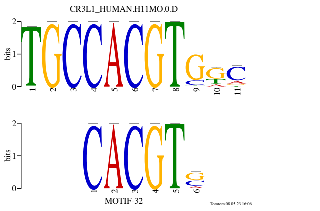

SRR1947691

FLI1

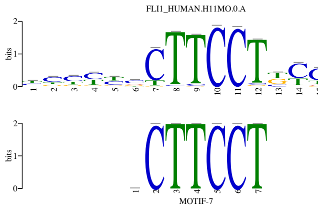

SRR1947692

ZN770

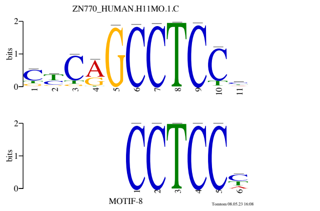

SRR1947693

CEBPZ

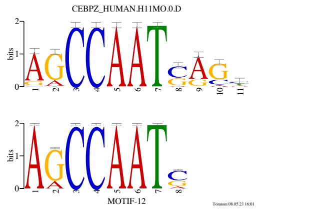

SRR2059127

NFYA

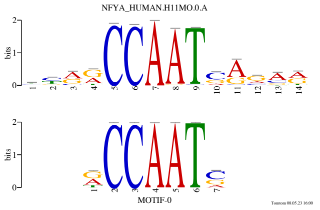

SRR2059128

CEBPZ

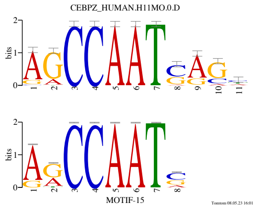

SRR2059129

CEBPZ

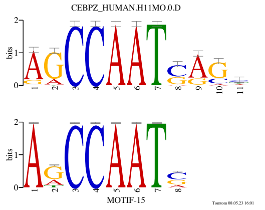

SRR2059131

NFYB

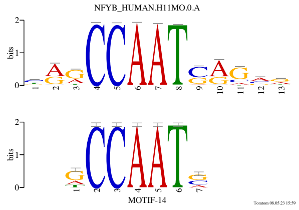

SRR2059151

CEBPZ

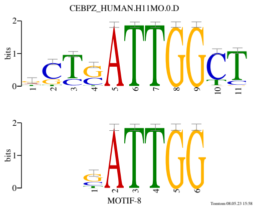

SRR2059152

NFYB

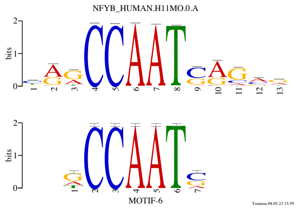

SRR2059153

ATF2

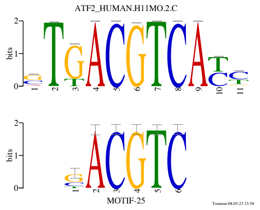

SRR2059154

MLX

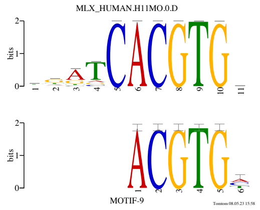

SRR2059156

CEBPZ

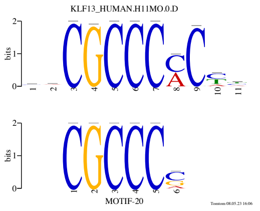

SRR2453159

NFYB

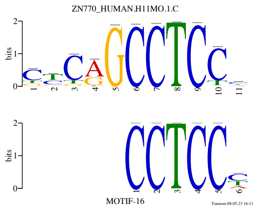

SRR2453161

ATF2

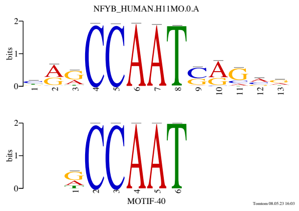

SRR2453162

MLX

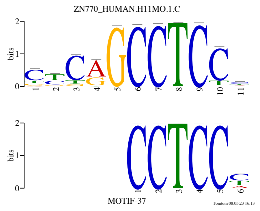

SRR2453163

ZIM3

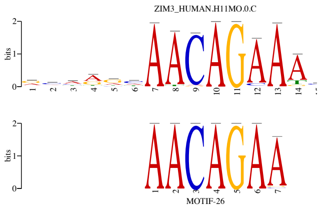

SRR2999315

ZBT14

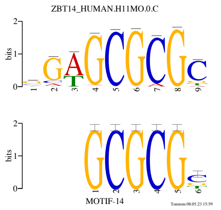

SRR3129116

ELF3

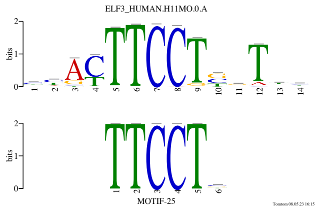

SRR4269916

ZN263

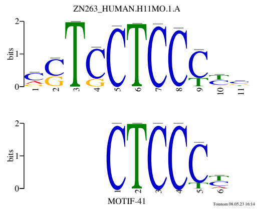

SRR4436441

ZN770

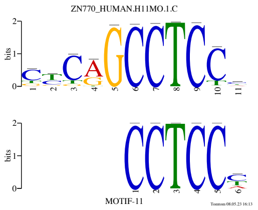

SRR5006007

ZN770

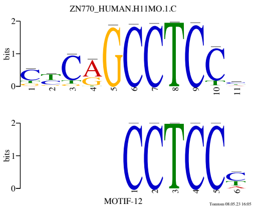

SRR5068860

NFYB

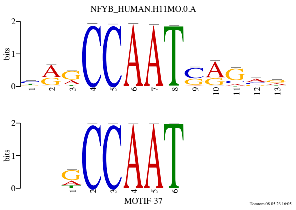

SRR5068861

NFYB

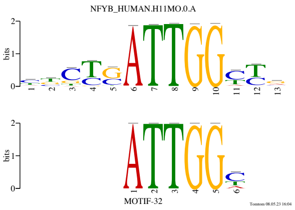

SRR5068863

ZN770

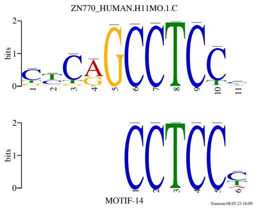

SRR5068865

MAZ

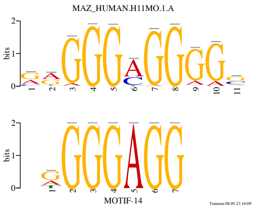

SRR5068866

GLIS2

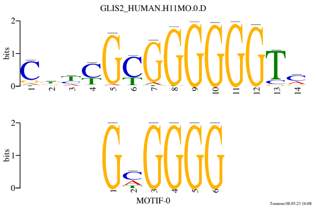

SRR5068867

ZN502

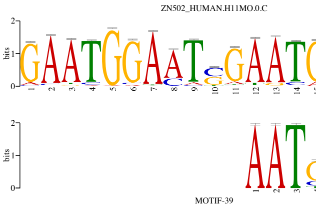

SRR5123142

ZIM3

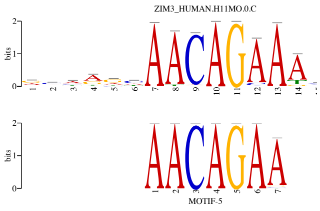

SRR5196903

OLIG1

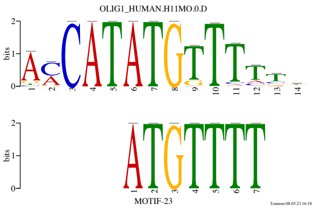

SRR5196904

ATF2

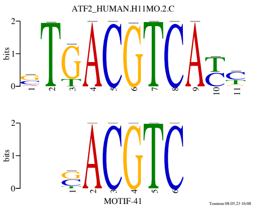

SRR5442269

MAZ

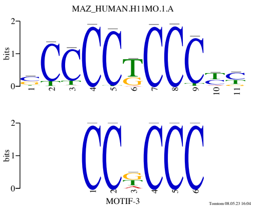

SRR5442270

GLI2

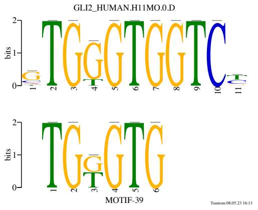

SRR5442271

MAFG

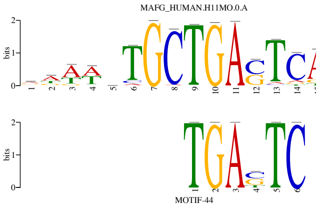

SRR5442275

ZN586

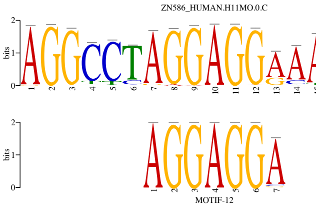

SRR5442276

NFYB

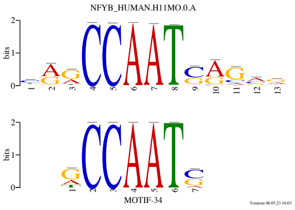

SRR5626533

HXB13

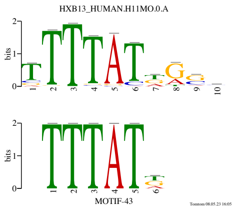

SRR5626534

NFYB

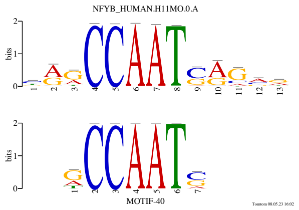

SRR5785357

MYF6

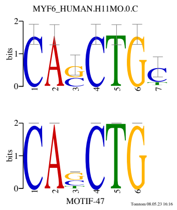

SRR5785364

ZSCA4

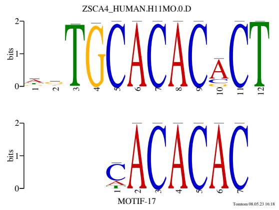

SRR5785369

FLI1

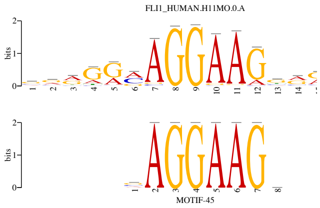

SRR5785370

NFYB

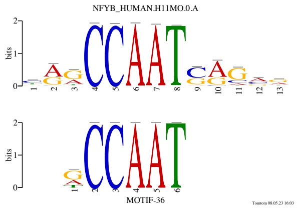

SRR5785377

ZN770

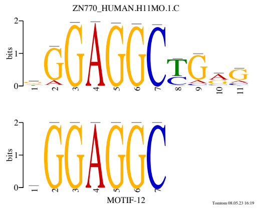

SRR5785386

FOSLI

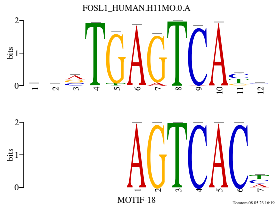

SRR5800664

MYF6

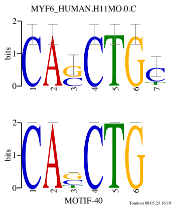

SRR5800665

ZN586

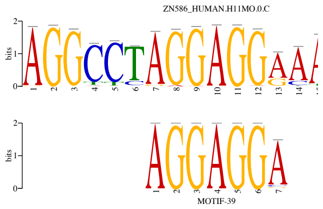

SRR5800706

SNAI2

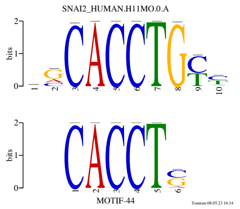

SRR5800797

ELF3

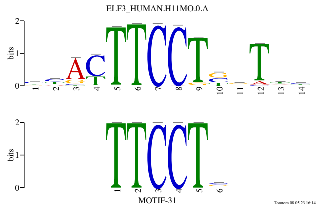

SRR5800799

MAZ

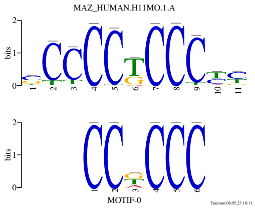

SRR5800801

GLI2

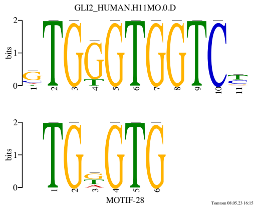

SRR5800802

ZN263

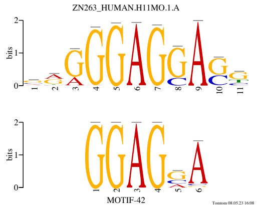

SRR6251841

ELF3

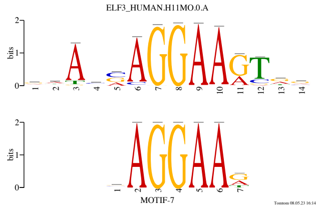

SRR6251843

ZN770

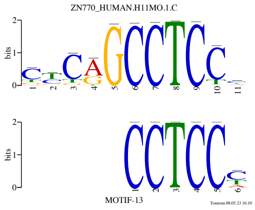

SRR6251844

MAZ

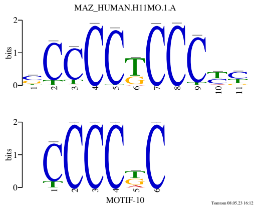

SRR6251845

FLI1

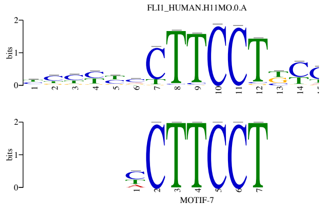

SRR6251846

ELF3

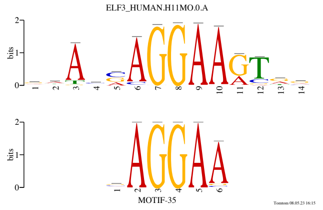

SRR6251848

ZN263

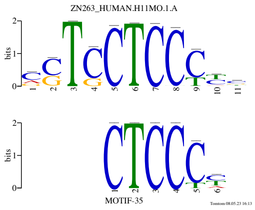

SRR6251849

ZN586

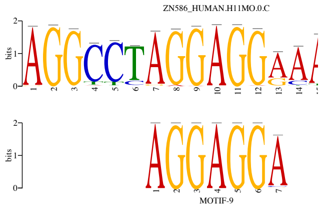

SRR6300362

MAZ

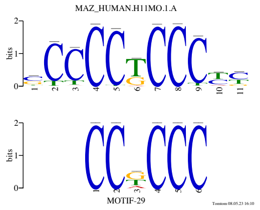

SRR6300363

ETV1

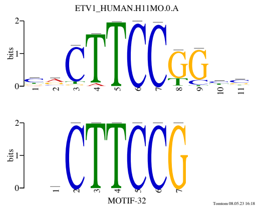

SRR6730131

ELF3

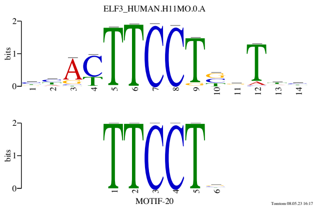

SRR6730132

ZN770

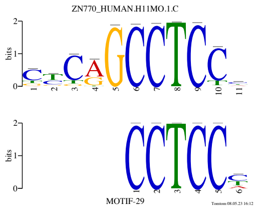

SRR6766910

MAZ

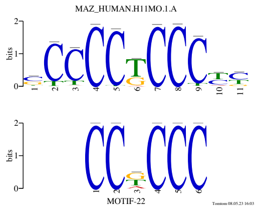

SRR6766911

MAZ

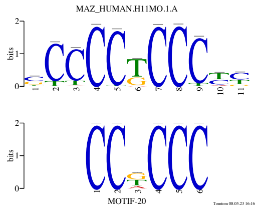

SRR6766912

ZN770

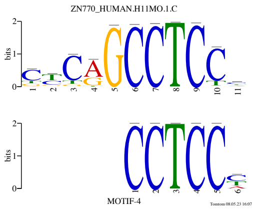

SRR6766913

ZN770

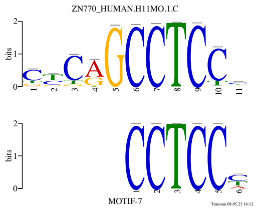

SRR6766914

MAZ

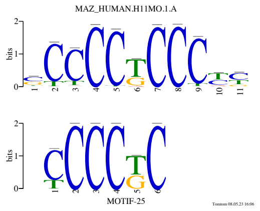

SRR6766915

CPEB1

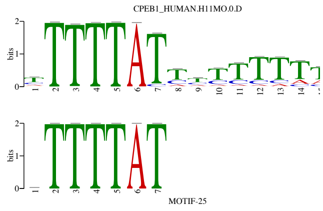

SRR6766916

ZN770

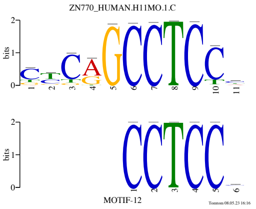

SRR6870516

ELF3

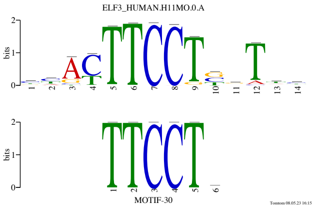

SRR6870517

ELF3

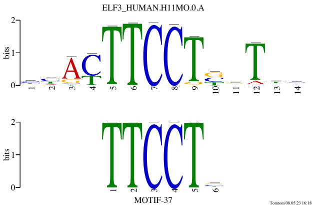

SRR6955605

MEIS3

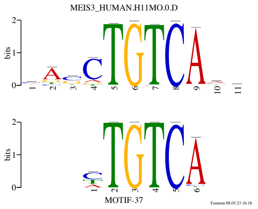

SRR6955608

ELF3

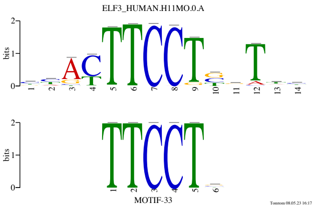

SRR7275228

ELF3

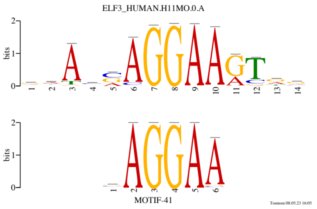

SRR1556325

ELF3

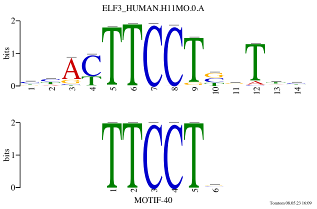

SRR1556326

ZN770

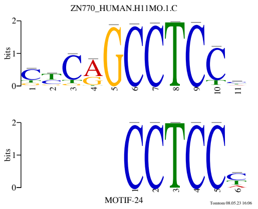

SRR1556327

FLI1

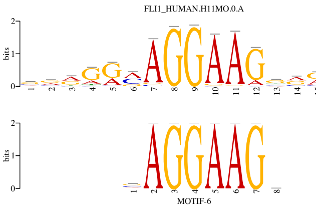

SRR1556329

ELF3

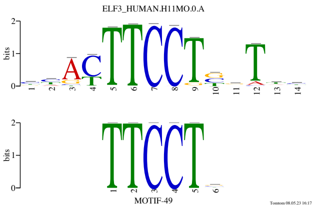

SRR1556330

ZN770

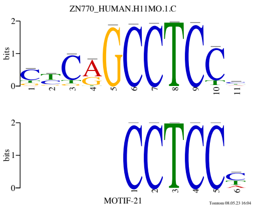

SRR1556331

ZN770

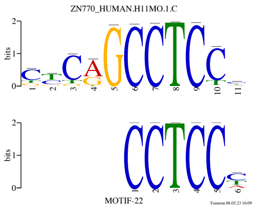

SRR1556332

SP1

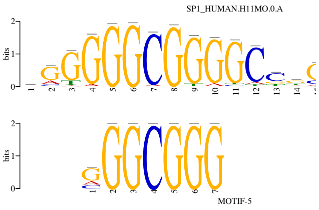

SRR1556333

SP1

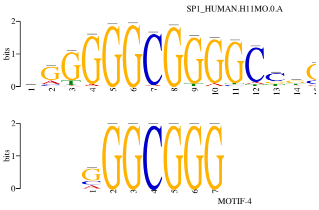

SRR1556334

NFYB

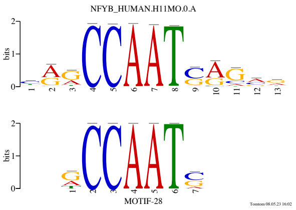

SRR1556335

CEBPZ

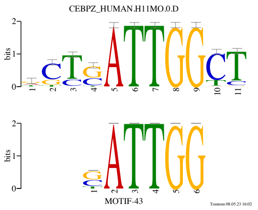

SRR1556336

NFYB

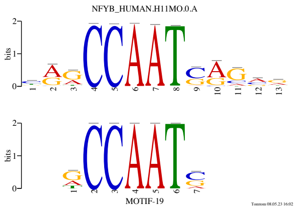

SRR1556337

MAZ

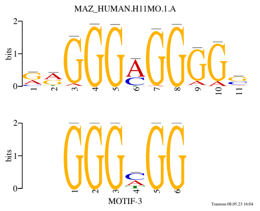

SRR1556338

ZN770

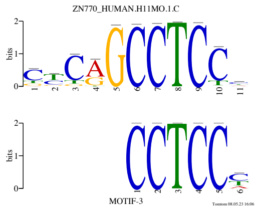

SRR1556339

ZN770

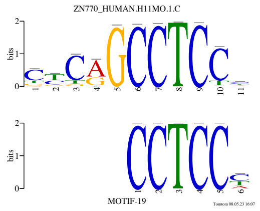

SRR1556340
